# Elevating surgical outcomes: a comprehensive analysis of innovative nursing practices in intraoperative care across diverse specialties

**DOI:** 10.3389/fmed.2025.1665160

**Published:** 2025-10-03

**Authors:** Ling Guo, Huanying Zuo, Yongmei Sun, Chengcheng Qian

**Affiliations:** ^1^Department of Anesthesiology and Perioperative Medicine, Shandong Provincial Hospital Affiliated to Shandong First Medical University, Jinan, China; ^2^Asthma Center, Children's Hospital Affiliated to Shandong University, Jinan, China; ^3^Department of Anesthesia and Surgery, Zibo Central Hospital, Zibo, China

**Keywords:** intraoperative nursing, surgical innovation, perioperative care, healthcare technology, patient outcomes

## Abstract

The landscape of surgical care is undergoing a profound transformation, driven by rapid advancements in technology and a persistent focus on enhancing patient outcomes. At the forefront of this evolution are innovative nursing practices within the intraoperative setting, which are proving instrumental in elevating patient safety, optimizing operational efficiency, and significantly reducing complications across a spectrum of surgical specialties. This report provides a comprehensive analysis of these cutting-edge nursing interventions from the foundational principles of perioperative care to the integration of advanced technologies such as Artificial Intelligence (AI), robotics, virtual reality, and personalized medicine. Perioperative nurses, traditionally recognized for their critical roles in maintaining sterile environments and advocating for patient wellbeing, are now embracing expanded responsibilities as technological integrators, data interpreters, and champions of evidence-based protocols such as Enhanced Recovery After Surgery (ERAS). The adoption of these innovative practices has led to demonstrable improvements, including shorter hospital stays, reduced opioid consumption, and decreased complication rates. However, the path to widespread adoption is not without its challenges, encompassing human factors such as resistance to change, organizational barriers such as insufficient resources, and technical complexities related to data accuracy and regulation. To fully harness the potential of innovative intraoperative nursing, healthcare organizations must strategically invest in continuous, accessible training programs that balance technological proficiency with core nursing judgment. Cultivating a culture that empowers nurse-led innovation, prioritizes human-centered technology design, and strengthens multidisciplinary collaboration is paramount. Furthermore, a commitment to evidence-based implementation and addressing health equity in technology access will ensure that advancements benefit all patients and that the nursing profession continues to thrive as a vital force in shaping the future of surgical care.

## 1 Introduction

Surgical care, a cornerstone of modern medicine, relies fundamentally on the meticulous and dynamic contributions of the intraoperative nursing team. As medical science and technology advance at an unprecedented pace, the role of these highly skilled professionals is continuously evolving, moving beyond traditional responsibilities to embrace innovative practices that directly influence patient outcomes (1, 2). This report delves into the transformative impact of these innovations, highlighting how perioperative nurses are not merely adapting to change but are actively driving improvements in patient safety, operational efficiency, and complication reduction across a diverse array of surgical specialties. Innovative nursing practices in intraoperative care are characterized by the proactive pursuit and development of novel methods, technologies, and tools aimed at promoting health, preventing diseases, and enhancing the overall quality of patient care (3). This extends beyond mere adoption of new tools; it involves critical thinking by nurses on the front lines, who are uniquely positioned to identify more efficient processes or repurpose existing items for alternate uses, thereby improving clinical practice (4). To ground this analysis, we acknowledge the foundational definition of the intraoperative nurse provided by organizations such as the Association of periOperative Registered Nurses (AORN) (5, 6). However, the role has expanded far beyond traditional duties. It is now defined by an internationally recognized competency profile that demands adept teamwork, sophisticated technology integration, and advanced critical decision-making to manage the complex, high-stakes surgical environment (7–10). These evolving competencies, which will be detailed further in this report, establish the intraoperative nurse not just as a caregiver but as a clinical leader and safety integrator. This perspective suggests that innovation is not a sporadic event or an optional add-on but rather a fundamental, ongoing requirement for contemporary nursing practice. For perioperative nursing to remain at the cutting edge and continuously improve patient care, fostering an environment where nurses are encouraged to actively seek and implement novel solutions is essential. This requires a shift in mindset, moving away from viewing innovation as an isolated project and toward embedding it as a core competency within nursing education and professional development programs from the outset. The observation that there is a lagging innovation ability among clinical nurses' points to a systemic gap that necessitates addressing through structured education and dedicated time for collaborative problem-solving and brainstorming (4, 11).

Perioperative nurses function as integral members of the surgical team, engaging in close collaboration with surgeons, anesthesiologists, and surgical technologists to deliver seamless and coordinated care (8, 9). During the intraoperative phase, their expertise is crucial in ensuring that all safety protocols are rigorously followed, that communication among team members is fluid and effective, and that patient rights and preferences are respected, particularly when the patient is under anesthesia and unable to self-advocate (3, 12). In this high-stakes environment, even with advanced technology, the unique human roles of vigilance, advocacy, and ethical oversight performed by nurses become even more critical. Nurses serve as a vital “human firewall” against potential errors, ensuring that technological advancements enhance care quality without diminishing the essential human element of patient safety and individualized attention. This highlights a crucial need for training programs that not only impart technological proficiency but also reinforce core nursing principles, ensuring a balanced approach to advanced surgical care.

This manuscript is a comprehensive narrative review designed to synthesize and analyze the existing literature on innovative nursing practices in intraoperative care. A structured literature search was conducted to identify relevant articles, guidelines, and reports.

## 2 Methodology

### 2.1 Search strategy and data sources

We searched several major electronic databases, including PubMed/MEDLINE, CINAHL (Cumulative Index to Nursing and Allied Health Literature), Scopus, and Google Scholar. The search was conducted to identify literature published between January 2010 and July 2025 to ensure a focus on contemporary and innovative practices. The search strategy employed a combination of keywords and Medical Subject Headings (MeSH) terms using Boolean operators (AND, OR). Key search terms included (“intraoperative nursing” OR “perioperative nursing” OR “theater nurse”) AND (“innovation” OR “technology” OR “advanced practice” OR “evidence-based practice”) AND (“surgical outcomes” OR “patient safety” OR “surgical efficiency”). Additional targeted searches were performed for specific topics, such as “Artificial Intelligence in surgery,” “robotics in nursing,” “Enhanced Recovery After Surgery (ERAS),” and “virtual reality in nursing education.”

### 2.2 Inclusion and exclusion criteria

Articles were selected for inclusion based on the following criteria:

- Inclusion criteria: the inclusion criteria were as follows: (1) peer-reviewed original research, systematic reviews, narrative reviews, and professional guidelines; (2) direct relevance to nursing roles and practices within the intraoperative phase of surgical care; (3) focus on the implementation or impact of new technologies, protocols (e.g., ERAS), or innovative nursing roles; and (4) published in the English language.
- Exclusion criteria: the exclusion criteria were as follows: (1) articles published before 2010; (2) studies focusing exclusively on preoperative or postoperative care without a clear link to intraoperative practices; (3) non-peer-reviewed content, dissertations, or conference abstracts without a subsequent full publication; and (4) articles not available in English.

### 2.3 Data synthesis

The selected literature was analyzed using a narrative synthesis approach. The information extracted from the articles was organized thematically according to the core topics of this review: foundational nursing principles, technological innovations [AI, robotics, virtual reality (VR)/augmented reality (AR), etc.], ERAS protocols, specialty-specific practices, and challenges to implementation. The findings were critically evaluated and integrated to construct a cohesive narrative that identifies key trends, quantifies impacts where data were available, and outlines future directions for intraoperative nursing. This approach allowed for a broad and comprehensive exploration of the topic from multiple perspectives.

## 3 The core competency profile of the contemporary intraoperative nurse

Contemporary intraoperative nursing is defined by a multifaceted competency profile that moves beyond foundational safety duties to integrate advanced clinical and systemic responsibilities. This profile, which serves as the standard for practice in today's technologically driven surgical environments, is understood through key domains encompassing complex teamwork, systems coordination, and technology management. The competencies detailed below demonstrate the evolution of the nurse's role from a task-oriented focus to that of a clinical leader and safety integrator (3, 6, 13, 14).

### 3.1 Foundational safety competencies

The bedrock of safe surgical practice is formed by several non-negotiable responsibilities: the meticulous setup and maintenance of the sterile field to prevent surgical site infections; the precise positioning of the client to prevent injury; and serving as the unwavering patient advocate to ensure safety and consent are verified when the patient is most vulnerable (15–18).

### 3.2 Teamwork and systems coordination

Intraoperative nursing excellence requires seamless integration within the multidisciplinary team. Nurses are central to facilitating clear and structured communication, a competency exemplified by the nurse-led implementation of standardized handoff protocols such as SHRIMPS to ensure continuity of care during personnel changes (1, 19). This extends to system-level coordination, where nurses play a pivotal role in implementing complex, evidence-based pathways such as ERAS. In this capacity, they coordinate actions across the entire surgical team to ensure adherence to protocols that accelerate recovery and reduce complications (20, 21).

### 3.3 Critical decision-making and evidence-based practice

**Figure 1:**
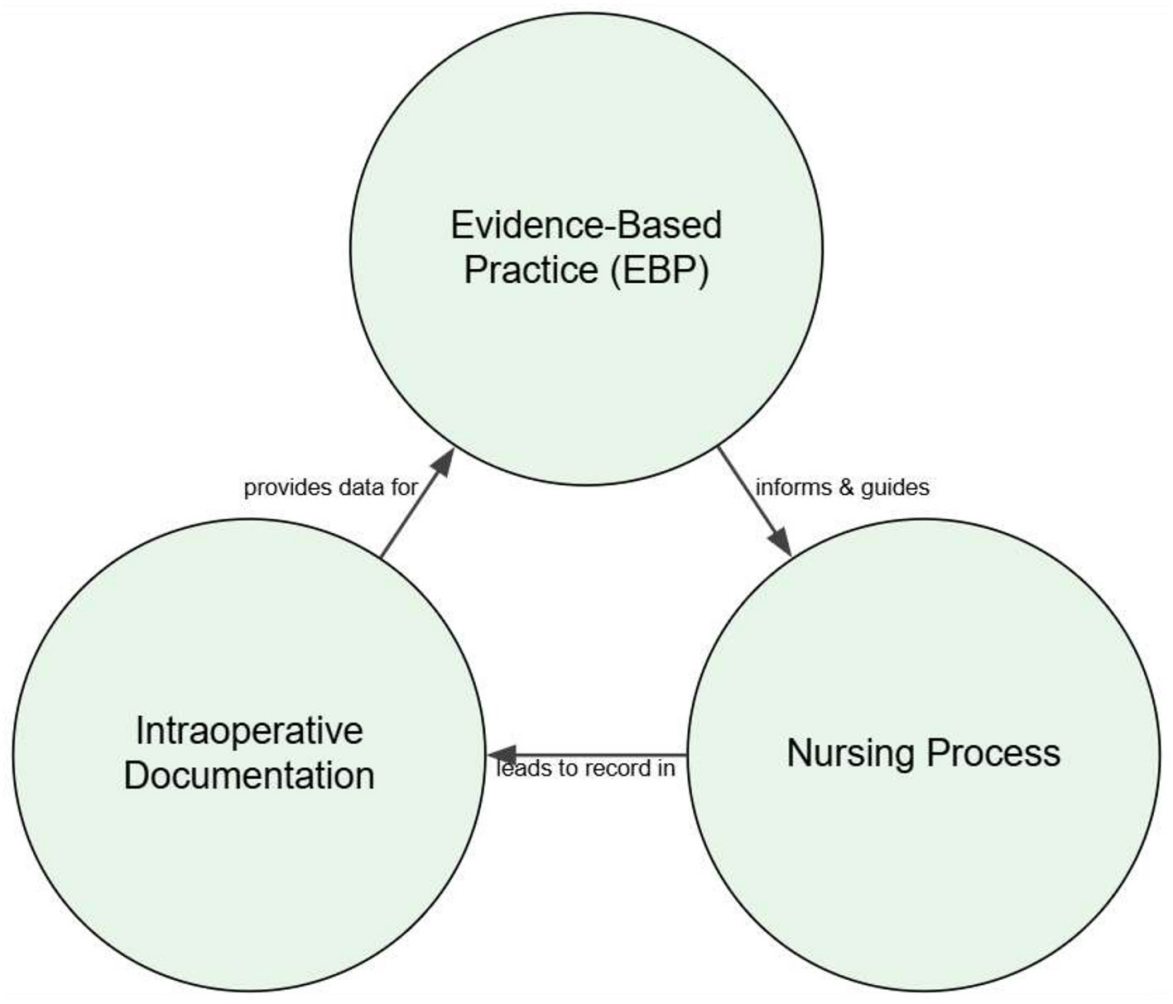
Dynamic framework for evidence-based intraoperative nursing. This model illustrates the cyclical and interdependent relationship between core nursing functions aimed at achieving optimal patient outcomes. The nursing process provides the structured methodology for patient care. This process is continuously informed by EBP, which integrates the best research to guide clinical decisions. All actions and observations are captured through intraoperative documentation, which not only ensures legal and safety standards but also provides critical data for quality improvement, feeding back into and refining future EBP. This cyclical flow highlights that intraoperative nursing is a continuous process of action, validation, and improvement.

The dynamic operating room environment demands constant critical thinking, which is structured through a clear and synergistic relationship between the nursing process, evidence-based practice (EBP), and documentation. The nursing process (assessment, diagnosis, planning, implementation, and evaluation) serves as the foundational framework for critical thinking, providing a systematic method for delivering individualized care (22, 23). This framework is then elevated by EBP, which informs every step of the process with the best available research, clinical expertise, and patient values (24). For example, when positioning a patient, the nurse uses the nursing process to assess risk, but it is EBP that guides them to select a specific, research-backed technique to prevent nerve damage (25, 26). Finally, meticulous intraoperative documentation serves as the indispensable record that captures the EBP-driven decisions made within the nursing process. This documentation provides legal proof of care, ensures continuity during handoffs, and supplies crucial data for quality improvement, making the entire cycle of thinking and action visible and verifiable (27, 28) (Figure 1).

### 3.4 Technology integration

A defining competency of the contemporary intraoperative nurse is the ability to manage and integrate advanced technologies. This role has shifted from manual assistance to that of a technology manager, responsible for preparing robotic systems, handling patient-specific 3D-printed guides, and interpreting data from an array of digital sources (29–31). As the subsequent sections will detail, this technological proficiency is not a separate skill but is woven into all other competencies to enhance surgical precision, improve efficiency, and elevate patient safety.

## 4 Technological innovations driving intraoperative care transformation

The integration of advanced technologies is fundamentally reshaping intraoperative nursing, moving beyond traditional methods to create more precise, efficient, and safer surgical environments. These innovations are not merely tools but are becoming integral partners in the delivery of patient care, demanding new skillsets and a collaborative approach from perioperative nurses (32) (Table 1).

**Table 1:** Impact of key technologies on intraoperative nursing outcomes.

**Technology**	**Primary impact on surgical outcomes**
Artificial intelligence	Reduced risk of complications through predictive alerts; improved OR efficiency.
Robotics	Enhanced surgical precision; reduced blood loss and shorter recovery times.
VR and AR	Improved nurse proficiency and confidence; decreased surgical error rates.
Wearable Health Tech	Earlier detection of patient deterioration; more personalized fluid management.
3D printing	Improved anatomical fit of implants; reduced operative time.
Blockchain	Enhanced data security and integrity; seamless continuity of care.

### 4.1 AI and robotics

AI and robotics are revolutionizing perioperative nursing by enhancing efficiency and clinical decision-making (33, 34). A key application of AI is predictive analytics, which uses machine learning algorithms to analyze vast datasets and forecast potential adverse events. This empowers nurses to move from a reactive to a proactive model of care by identifying high-risk patients before complications arise (35). In practice, AI offers real-time decision support during surgery and helps optimize OR logistics such as scheduling and staff assignments, freeing nurses to focus on direct patient care (36–38).

Robotic systems, often guided by AI, enhance surgical precision, minimize invasiveness, and reduce the administrative burden on nursing staff (39). For nurses, the integration of these technologies marks a shift from manual assistance to the role of a technology manager. Key responsibilities now include preparing robotic equipment, interpreting data from predictive models, and applying critical thinking to ensure that all technological recommendations align with the patient's holistic needs (36, 39).

### 4.2 Virtual reality (VR) and augmented reality (AR)

VR and AR are transforming nursing education and patient experience. These immersive technologies provide realistic, risk-free training simulations, allowing nurses to practice complex procedures and enhance their clinical reasoning (40, 41). For patients, VR can be used to reduce preoperative anxiety and manage postoperative pain (42, 43). AR provides real-time information overlays during procedures, aiding in anatomical visualization and procedural guidance (44). The primary impact is improved nursing proficiency and enhanced patient safety through better training and a reduction in surgical errors (45).

### 4.3 Wearable health technologies

Wearable health technologies are creating a new continuum of patient data that directly impacts intraoperative nursing practice. While traditionally used for pre- and postoperative monitoring, these data provide a crucial baseline for the intraoperative period. The nurse can access a patient's pre-operative data from their personal wearable devices—such as activity levels, sleep patterns, or baseline heart rate variability—to create a more holistic and personalized intraoperative care plan (46). Furthermore, the emergence of intraoperative wearable sensors is creating a new stream of real-time data for which the nurse is responsible. These sensors can offer continuous monitoring of core body temperature, muscle oxygenation, or subtle hemodynamic shifts that may not be captured by standard monitors. The intraoperative nurse's role is to integrate this new data with traditional monitoring, interpret its clinical significance, and use it to anticipate needs and facilitate timely interventions (47–49). This transforms the nurse's function from periodic data collection to the continuous, proactive surveillance of a data-rich patient, directly connecting wearable technology to intraoperative safety and outcomes (31, 50–52).

### 4.4 3D printing

3D printing enables the creation of patient-specific surgical guides and implants, shifting a portion of the surgical planning to the pre-operative phase (53–55). However, this innovation places a critical and high-stakes responsibility on the intraoperative nurse, who functions as the final point of safety verification within the sterile field. The nurse's specific contribution involves more than just instrument management; it is a crucial safety intervention. They are responsible for meticulously cross-referencing the unique identifiers on the sterile 3D-printed device against the patient's records and the surgical plan immediately before use. By confirming this match and communicating it to the team, the nurse provides a definitive safeguard against a potential wrong-site, wrong-procedure, or wrong-implant error. This direct nursing action is indispensable for the safe and effective application of personalized surgical technology, directly linking their vigilance to improved surgical precision and patient outcomes (56–58).

### 4.5 Blockchain technology

Blockchain technology enhances the security, integrity, and privacy of patient data by providing a decentralized and immutable ledger for health information (59, 60). For perioperative nurses, this means becoming familiar with advanced charting and documentation systems that leverage blockchain principles, ensuring that data are accurately recorded and securely accessible for continuity of care (61) (Table 1).

## 5 Enhanced recovery after surgery (ERAS) protocols: a nursing imperative

ERAS protocols represent a paradigm shift in perioperative management, moving from traditional, fragmented care to a holistic, evidence-based, and patient-centered approach. These multimodal pathways are designed to reduce the physiological and psychological stress of surgery, thereby accelerating recovery and improving outcomes (62). Far from being a simple checklist, ERAS is a dynamic process where nurses play a central, coordinating role across the entire surgical journey (20).

### 5.1 The nurse's role across the ERAS continuum

The success of ERAS is critically dependent on nursing leadership and meticulous execution of specific interventions at each phase (20, 21, 63):

1. Preoperative phase: the nurse's role begins well before surgery with crucial interventions such as patient education and counseling, nutritional optimization through protocols like carbohydrate loading, and promoting pre-habilitation (64, 65).
2. Intraoperative phase: inside the operating room, the intraoperative nurse is key to maintaining the physiological balance that is central to ERAS. Their specific contributions include the following (66–68):
  ○ Goal-directed fluid therapy: collaborating closely with the anesthesia team to maintain euvolemia (optimal fluid balance), preventing both dehydration and fluid overload, which are known to cause complications.
  ○ Normothermia maintenance: actively managing patient temperature using warming blankets and other technologies to prevent inadvertent hypothermia, which can impair wound healing and increase infection risk.
  ○ Opioid-sparing multimodal analgesia: working with the team to utilize a combination of non-opioid analgesics and regional anesthesia techniques, a cornerstone of ERAS is designed to reduce opioid-related side effects such as nausea and ileus.
3. Postoperative phase: the nurse's role is perhaps most visible in the postoperative phase, where they drive the recovery process through (63, 69):
  ○ Early mobilization: actively encouraging and assisting patients to get out of bed and walk within hours of their surgery to prevent blood clots, improve lung function, and hasten the return of bowel function.
  ○ Early oral nutrition: promptly discontinuing IV fluids and reintroducing the oral diet to stimulate gut function and provide necessary nutrients for healing.
  ○ Proactive nausea and vomiting management: administering antiemetics prophylactically to prevent these common side effects, which are major barriers to early feeding and mobilization.

### 5.2 Impact and nursing leadership

The implementation of these nurse-driven ERAS interventions has yielded significant, quantifiable improvements in patient outcomes. Studies consistently show reduced length of hospital stay (LOS), decreased complication rates, and a dramatic reduction in opioid consumption (70, 71).

ERAS empowers nurses by positioning them as leaders and coordinators of the multidisciplinary team. It demands a high degree of autonomous clinical judgment and challenges traditional hierarchies, requiring strong institutional support to overcome resistance to changes such as early feeding or catheter removal (72). Ultimately, ERAS is a powerful example of how structured, evidence-based nursing practice can fundamentally elevate the standard of surgical care.

## 6 Innovative organizational models: staffing based on nursing care complexity

Beyond individual practices, significant innovation occurs at the organizational level through new models of perioperative nursing care. Traditional staffing approaches, often based on rigid, one-size-fits-all ratios, are being replaced by more dynamic, evidence-based frameworks (73, 74). Driven by pressures such as staff shortages and the need for greater efficiency, these models prioritize flexibility and the strategic alignment of nursing competencies with specific patient and procedural demands (75).

The central principle of these innovative models is the shift from staffing based on surgical procedure type to a more nuanced allocation based on nursing care complexity. This involves a data-driven assessment of the anticipated nursing workload, recognizing that a technically simple surgery may still require intensive nursing care and vice versa (76). Key features of these models often include the strategic use of flexible roles, such as a circulating nurse who can support multiple operating rooms, and the empowerment of nurses through their involvement in workload assessment and scheduling decisions (76, 77). This aligns with frameworks such as the WHO's Workload Indicators of Staffing Need (WISN), which advocate for staffing based on empirical data rather than tradition (78).

A practical application of these principles is demonstrated by Cenacchi et al. (77), who implemented a tiered staffing model at a major university hospital. By differentiating between surgical and nursing complexity, they were able to create a flexible system: high-intensity nursing procedures were assigned three dedicated nurses, while medium-intensity cases were managed effectively with fewer staff by incorporating a shared circulating nurse. This data-driven approach allowed the organization to optimize resource allocation, ensuring that expertise was concentrated where it was most needed (77).

Ultimately, these innovative organizational models enhance operational efficiency and patient safety while fostering greater professional autonomy and leadership within the intraoperative nursing team.

## 7 Innovative nursing practices across diverse surgical specialties

Innovations in intraoperative nursing are manifesting at both the broad organizational level and in the specialized, hands-on practices within different surgical domains. A holistic view reveals a shift toward more dynamic, evidence-based systems that empower nurses to enhance patient safety and efficiency.

### 7.1 Specialty-specific adaptations of intraoperative nursing practice

Within these innovative organizational structures, the core competencies of intraoperative nursing are adapted with specialized clinical reasoning to meet the unique demands of each surgical field. The nurse's role is not static; it requires dynamic application of skills and knowledge tailored to the specific technologies, procedures, and patient risks of the specialty.

#### 7.1.1 Cardiac surgery

The nurse's role in this high-acuity environment centers on the precise management of sterile components for life support. A primary responsibility is handling the aortic and venous cannulas for cardiopulmonary bypass (CPB), ensuring correct sutures are available for securing them, and managing the cardioplegia delivery system used to arrest the heart. The nurse collaborates directly with the perfusionist by handling the sterile tubing and probes passed to and from the CPB machine. They must also manage multiple chest tubes and epicardial pacing wires, anticipating the surgeon's needs with specialized long instrumentation for deep thoracic work, all while contributing to the meticulous management of the patient's coagulopathy and hemodynamic stability (79–85).

#### 7.1.2 Orthopedic surgery

Nursing practice here is defined by the management of high-torque equipment and complex implant sequences. In joint arthroplasty, the nurse's role involves the sequential organization of numerous reamers, broaches, and trials, ensuring the correct size is available at each step. For robotic-assisted procedures, specific nursing tasks include sterile draping of the robotic arm, assisting with the anatomical registration process, and managing the robotic end effectors. Furthermore, the nurse is responsible for the precise mixing and handling of polymethylmethacrylate (PMMA) bone cement, a critical step that involves being vigilant for the signs of Bone Cement Implantation Syndrome (BCIS) and managing the cement's exothermic reaction (86–91).

#### 7.1.3 Neurological surgery

The nurse in neurosurgery acts as a direct guardian of neurological tissue through meticulous technique. Key responsibilities include sterile draping and intraoperative manipulation of the surgical microscope, which requires delicate coordination with the surgeon. In procedures utilizing Intraoperative Neuromonitoring (IONM), the nurse's specific contribution involves managing the sterile field to prevent interference with monitoring signals; this includes carefully placing subdermal needle electrodes as directed and controlling saline irrigation to avoid creating electrical bridges that could corrupt the data. Constant management of irrigation fluids is also critical for maintaining the temperature and moisture of delicate neural structures, directly helping to prevent iatrogenic injury (22, 23, 92–94).

#### 7.1.4 General surgery (laparoscopic)

In minimally invasive surgery, the nurse functions as a technology and system specialist. Their role extends beyond handling instruments to managing the entire laparoscopic setup from the sterile field. This includes troubleshooting the camera (e.g., cleaning, white-balancing, and defogging the lens) and managing the pneumoperitoneum by communicating with the anesthesia provider about insufflation pressures. A critical safety function is managing multiple advanced energy devices (e.g., ultrasonic scalpels and vessel sealers). The nurse must ensure each device is connected to the generator with the correct power settings requested by the surgeon, a crucial step in preventing unintended thermal injury to the patient (1, 19, 95, 96) (Table 2).

**Table 2:** Innovative nursing practices across diverse surgical specialties.

**Surgical specialty**	**Primary competency applied**
Cardiac surgery	Systems coordination & critical decision-making.
Orthopedic surgery	Technology integration & foundational safety.
Neurological surgery	Technology integration & teamwork/communication.
General surgery	Technology integration & evidence-based practice.

### 7.2 Synthesis: key themes in nursing innovation

While the literature extensively details technological innovations within various surgical specialties, there is a noticeable gap in explicitly detailing the nuanced nursing roles developed specifically for the intraoperative phase (93). The precise contributions of nurses in integrating technologies such as robotics and 3D printing often remain implicit rather than explicitly documented (1, 97). This underscores the critical importance of dedicated nursing research to articulate and validate these unique and often “unsung” contributions, ensuring that the full scope of nursing innovation is recognized.

Despite the increasing technological sophistication across different surgical fields, a consistent and profound theme remains: the unwavering commitment to holistic patient care, encompassing both physical and psychological wellbeing (98, 99). The humanistic aspects of nursing—providing comfort, managing anxiety, and addressing patient fears—remain a constant and innovative imperative. This highlights that technological progress must always be balanced with compassionate, comprehensive care to achieve the best possible patient outcomes (97, 100–102).

## 8 Challenges and strategies for innovation adoption in perioperative nursing

The integration of innovative practices and advanced technologies into perioperative nursing, while promising, is often met with a complex array of challenges. These obstacles span human, organizational, and technical domains, necessitating comprehensive and strategic approaches for successful and sustainable adoption (1, 103).

### 8.1 Barriers

- Human factors: resistance to change is a significant barrier, often stemming from skepticism among nurses who fear job displacement or punitive use of data generated by new technologies (104, 105). Nurses, who rely heavily on intuition and clinical judgment, may be concerned that AI or other automated systems could diminish their professional autonomy and expertise (106). A lack of understanding of the benefits of new innovations and a fear of technology failures further contribute to this resistance (100, 107).
- Organizational/systemic factors: healthcare organizations frequently face systemic barriers to innovation. These include a lack of sufficient time for training and adaptation to new systems, inadequate funding for technology acquisition and implementation, and insufficient institutional support for innovative initiatives (4). High employee turnover and excessive workload also hinder the capacity for nurses to engage with and adopt new practices (108). A notable challenge is the lack of uniform engagement with new protocols, such as ERAS, across multidisciplinary teams, leading to inconsistencies in care (72). Furthermore, the haphazard adoption of new surgical technologies without proper evaluation carries the potential for significant patient harm, as evidenced by past experiences (109).
- Technical/data factors: technologies themselves can present barriers. Concerns about the accuracy and reliability of data used to train AI tools, a lack of clear regulation and transparency in AI algorithms, and critical issues related to patient privacy and data security are prevalent (101, 110). The inherent complexity of integrating new technologies can also disrupt existing workflows, causing delays and resistance (107, 111).

The implementation of innovative nursing practices and technologies in the operating room is fundamentally a change management challenge. Technical solutions alone are insufficient; success hinges on addressing the psychological, cultural, and systemic factors that influence human behavior and organizational readiness. This means that strategic planning for innovation must prioritize human-centered design, robust training, and empowering frontline nurses as co-creators and champions of change (Table 3).

**Table 3:** Barriers and facilitators to technology adoption in perioperative nursing.

**Category**	**Specific barriers**	**Key facilitators/strategies**
Human/psychological	Resistance to change, skepticism (fear of replacement, punitive use), lack of understanding of benefits, fear of technology failures, over reliance on technology eroding critical thinking (142)	Fostering a culture of change and experimentation, clear communication of “the why,” empowering staff, promoting divergent thinking, emphasizing AI as a decision-support tool (143).
Organizational/systemic	Lack of time for training/adaptation, insufficient funding, inadequate institutional support, high employee turnover, excessive workload, lack of uniform engagement across teams, haphazard adoption (4)	Flexible frameworks, phased rollouts, adequate resource allocation, strong nursing leadership involvement, multidisciplinary collaboration, continuous auditing and feedback (144).
Technical/data	Data accuracy and reliability issues in AI tools, lack of regulation and transparency in AI, patient privacy/data security concerns, complexity disrupting workflows (114)	Ensure AI tools are evidence-based, focus on empowerment not surveillance, involve nurses in AI implementation, ensure data protection, provide ongoing support and training for usability (145).

### 8.2 Critical appraisal of ethical dilemmas in technology integration

The integration of advanced technologies into intraoperative care presents profound ethical challenges that extend beyond technical implementation to the core of nursing practice. While frameworks such as the ANA's “5 Rights of AI” offer guidance (112), a deeper critical appraisal is necessary to navigate the complex dilemmas nurses face. This requires operationalizing core bioethical principles—beneficence (acting for the patient's good), non-maleficence (do no harm), and justice (ensuring equitable care)—within the high-stakes surgical environment.

Concrete ethical dilemmas frequently emerge in practice. For example:

- Algorithmic bias and justice: consider an AI-driven decision support tool that uses historical data to predict a patient's risk of surgical site infection. If the algorithm was trained on data that underrepresents certain demographic groups, it may inaccurately assign a lower risk score to a patient from that group, leading to less stringent intraoperative precautions. This creates a direct conflict with the principle of justice. The nurse is faced with the dilemma of either following the flawed data-driven recommendation or advocating for a higher level of care based on their own assessment, potentially challenging the protocol (113).
- Accountability and clinical judgment: a critical conflict arises when an AI-generated alert or recommendation contradicts an experienced nurse's clinical judgment (106). If a wearable device shows stable vitals but the nurse observes subtle, unquantifiable signs of patient deterioration, a decision must be made. If the nurse trusts their intuition and initiates a rapid response, they may be questioned for acting against the data. If they trust the technology and a negative outcome occurs, who bears the responsibility? This ambiguity in accountability—whether it lies with the nurse, the institution, or the technology developer—is a significant unresolved challenge.
- Data privacy and autonomy: the continuous stream of data from integrated technologies creates vulnerabilities. A data privacy breach could expose highly sensitive patient information, violating the principle of non-maleficence by causing potential harm (114). Furthermore, ensuring true informed consent (patient autonomy) becomes more complex when patients may not fully understand how their data are being used, stored, or potentially commercialized.

Operationalizing an ethical response requires nurses to act as vigilant patient advocates. This involves treating AI tools as adjuncts, not arbiters, of care; questioning and reporting potential biases in technology; and championing robust data security measures. The nurse's role is to serve as the essential “human firewall,” ensuring that technological advancements are always applied through a lens of ethical reasoning, clinical expertise, and unwavering commitment to patient safety and equity (115, 116).

### 8.3 Strategies for successful implementation

Overcoming these barriers requires a multi-faceted and integrated approach:

- Fostering a culture of change and innovation: healthcare organizations must actively cultivate an environment that embraces change and innovation. This involves encouraging experimentation, reframing failures as valuable learning opportunities, and empowering staff to actively contribute to continuous improvement initiatives (117, 118). Promoting divergent thinking, encouraging calculated risk-taking, fostering tolerance for failure, and building agility and autonomy within nursing teams are crucial steps in this cultural transformation (119, 120).
- Flexible frameworks and phased rollouts: a “one-size-fits-all” approach to innovation is often ineffective. Instead, strategies should be customized based on the unique needs of each unit, patient population, and acuity level (119). Implementing a test-and-launch model, starting with small-scale pilots and gradually expanding, can effectively reduce risk and increase proper usage and acceptance (121).
- Targeted education and simulation-based training: comprehensive education is essential for successful innovation adoption, and simulation-based training stands out as a key strategic facilitator. This approach allows nurses to develop and refine skills in a safe, controlled environment without any risk to patients. These programs should focus not only on the technical operation of new technologies such as robotics or VR but also on critical thinking, teamwork, and crisis management skills required to use them effectively (122, 123). High-fidelity simulations, whether using advanced mannequins or immersive Virtual Reality (VR) environments, bridge the gap between theory and practice (102, 124). They enable nurses to build muscle memory for complex procedures, practice communication and decision-making during rare or high-stakes events, and gain confidence with new technologies, which helps overcome resistance to change and fear of failure (40, 41). By integrating robust simulation into training, healthcare organizations can ensure that intraoperative nurses achieve both technical proficiency and the situational awareness needed to translate innovation into safer patient care (125).
- Strong nursing leadership and multidisciplinary collaboration: early involvement of nursing leaders in the decision-making process for technology selection and implementation is paramount (126). Nurse leaders are uniquely positioned to encourage, motivate, and empower their colleagues and other healthcare professionals to embrace new practices (72, 127). Building cohesive multidisciplinary teams that are aligned and “pushing in the same direction” is critical for a successful implementation of complex protocols such as ERAS (128, 129). Adherence to professional guidelines from organizations such as AORN, ANA, and WHO provides a foundational framework for safe and effective innovation (7, 112, 130, 131).
- Communicate “the why”: clearly articulating the purpose and tangible benefits of new tools to clinicians is crucial for gaining acceptance and buy-in. Utilizing multiple communication channels to reinforce these messages ensures broader understanding and engagement (132, 133).

### 8.4 The imperative of evidence-based implementation for sustainable innovation

While innovation is crucial for advancing healthcare, its adoption must be rigorously guided by evidence and an unwavering commitment to patient safety. The literature highlights that “haphazard adoption of new surgical technologies without proper evaluation has the potential to cause significant harm” (109). Therefore, nurses are advised to consider innovation “in the context of evidence-based practice” (134, 135). A significant challenge lies not only in developing innovations but also in systematically evaluating their efficacy and integrating them into practice through robust evidence-based implementation strategies. This is particularly relevant for protocols such as ERAS, where, despite an “extensive evidence-base,” nurses may still hesitate to fully implement elements without explicit clinician “permission” due to ingrained traditional mindsets (72, 136). This situation underscores the need for strong leadership to champion evidence-based approaches, overcome resistance to change based solely on tradition, and ensure that new practices are not just adopted but are *sustained* through continuous auditing and feedback loops. This ensures that innovation truly translates into improved patient outcomes and becomes a permanent feature of perioperative care.

## 9 Conclusion, future directions, and recommendations

The comprehensive analysis presented in this report unequivocally demonstrates that innovative nursing practices are profoundly elevating surgical outcomes across diverse specialties. The integration of advanced technologies, coupled with a steadfast adherence to evidence-based protocols like ERAS, has consistently led to enhanced patient safety, improved operational efficiency, and significant reductions in complications. Perioperative nurses, once primarily focused on technical assistance, have evolved into indispensable leaders within the surgical team. Their roles now encompass sophisticated technological integration, astute data interpretation, and the championing of holistic, patient-centered care. This evolution underscores the critical importance of the perioperative nurse as a technologically adept, critically thinking, and ethically grounded advocate, whose contributions are essential for optimizing patient experiences and achieving superior surgical results.

### 9.1 Strategic imperatives for the future operating room

The integration of AI and advanced technologies is redefining the perioperative nurse's role from a technical assistant to a clinical data curator and technology manager (137, 138). This shift requires a proactive response from healthcare organizations. To ensure nurses can meet these future demands, institutions must invest in comprehensive, continuous training programs. These programs must extend beyond technical operation to include data literacy, critical evaluation of AI outputs, and the ethical implications of new technologies (139). Simultaneously, organizations must foster a culture of nurse-led innovation by providing the time, resources, and institutional support for nurses to lead the change (117, 118). True integration also demands strengthening multidisciplinary collaboration through standardized communication protocols and shared technological platforms that ensure seamless teamwork across the entire surgical journey.

### 9.2 The ethical mandate for equitable and human-centered care

As surgical care becomes highly personalized through genomics and data-intensive technologies, profound ethical challenges emerge regarding equitable access, data privacy, and the potential for algorithmic bias to worsen health disparities (140, 141). Nursing profession's core commitment to patient advocacy mandates an ethically grounded approach to technology adoption (115, 116). Therefore, organizations must develop and implement clear ethical frameworks for all advanced technologies, ensuring that patient privacy, data security, and human accountability remain paramount. As patient advocates, nurses should be central to the development and review of these frameworks. Furthermore, there is an imperative to address health equity by creating strategies that ensure advancements in surgical care and training are accessible to all patients and clinicians, including those in rural or under-resourced settings. Ultimately, technology adoption must be prioritized through a human-centered lens, involving nurses in the design and selection of tools to guarantee they augment, rather than impede, the essential clinical judgment and compassionate care that define the nursing profession.
